# Microsatellite Variation of African Elephants *Loxodonta africana* Blumenbach 1797 in a Savannah Protected Area of South Sudan

**DOI:** 10.1002/ece3.71383

**Published:** 2025-06-05

**Authors:** Thomas Francis Lado, Wani Felix Jibi, Pasquale Tiberio Moilinga

**Affiliations:** ^1^ Department of Wildlife Science, College of Natural Resources and Environmental Studies University of Juba Juba South Sudan

**Keywords:** bottleneck, genetic diversity, inbreeding, relatedness, savannah elephant *Loxodonta africana* Blumenbach 1797

## Abstract

Anthropogenic activities such as poaching and habitat loss lead to a drop in population size, range overlap and hybridisation. The decline in population size results in reduced genetic diversity, an increase in homozygosity and inbreeding. Here, we genotyped 16 polymorphic microsatellite loci on 80 elephant dung samples to determine genetic diversity, genetic bottleneck, genetic relatedness and inbreeding in the savannah elephant in Nimule National Park, which experienced an 80% fall in population size. Results revealed that the elephant population in the park comprised 26 savannah elephants. The study also found genetic variation, average number of observed alleles A_o_, observed heterozygosity H_o_ and expected heterozygosity H_e_ to be 5.31 ± 2.62, 0.61 ± 0.22 and 0.56 ± 0.21, respectively, but with no difference between observed H_o_ and expected H_e_ heterozygosity. There was no evidence that the elephant population in the park went through a recent genetic bottleneck (*p* = 0.94167; and normal L‐shaped distribution); however, evidence for a historical bottleneck was detected (M ratio = 0.44 ± 0.22). Mean pairwise relatedness was generally low (ML‐*r* = 0.09 ± 0.22) with a high proportion of unrelated individuals (U = 85.8%), and there was no indication of inbreeding (F_IS_ = −0.08, *p* > 0.05). We conclude that the observed decline in the population size is not an artefact of using different methods, as shown by the historical bottleneck. Despite the observed reduction in census size, there is an exchange of individuals with the neighbouring savannah elephant population.

## Graphical abstract

Poaching and habitat loss reduce population size, genetic diversity, genetic bottleneck, genetic relatedness and inbreeding in the savannah elephant population.

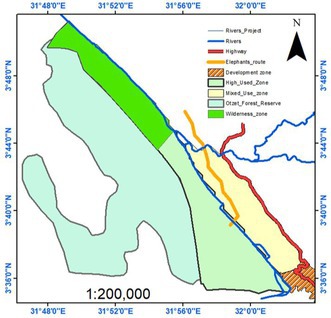

## 1 Introduction

Many endangered species are threatened by habitat loss and fragmentation, illegal hunting, climate changes, diseases, pollution and other natural calamities which further contribute towards population decrease thereby leading to local extinction (Girod et al. 2011). Population decline can lead to genetic drift due to genetic bottleneck (Frankham et al. 2010) and loss of genetic diversity (Bouzat 2010; Nei et al. 1975; Tajima 1989, 1996), and inbreeding (Frankham et al. 2010; Freeland et al. 2011). A severe reduction in population size may result in few individuals being left and may, as such, enhance nonrandom mating between individuals, thus resulting in inbreeding depression (Bryant et al. 1999; Charlesworth and Charlesworth 1987; Hedrick 1987; Packer et al. 1991). Emigration into other populations in search of refuge may result in population admixture, subsequently providing favourable conditions for hybridisation, especially for related species (Frankham et al. 2010; Freeland et al. 2011). Loss of genetic diversity, exemplified by loss of alleles, can increase homozygosity (Beebee and Rowe 2008; Frankham et al. 2010). It will also affect the ability of a species to cope with changes in the environment and to resist diseases (Frankham et al. 2010; Freeland et al. 2011). Increased levels of relatedness in wildlife populations and species can lead to the loss of genetic variation due to mating between close relatives and to random genetic drift (Frankham et al. 2010; Keller and Waller 2002). This can result in increase in the expression of recessive deleterious alleles, the loss of heterozygosity and the extinction of functionally important alleles in the population (Frankham et al. 2010; Keller and Waller 2002). A surge in homozygosity can culminate in inbreeding, hence reducing the fitness of an individual, which, when coupled with genetic drift, can drive certain alleles to fixation or extinction in a short time (Frankham et al. 2010; Freeland et al. 2011).

The African elephant, *Loxodonta africana* Blumenbach 1797, is the largest extant terrestrial mammal in Africa (Stuart and Stuart 1998). It takes long to reach reproductive maturity and mate, and has a low intrinsic rate of reproduction of about 7% (Calef 2008) and thus is especially susceptible to poaching (Stuart and Stuart 1998). The species has been overhunted mainly to satisfy the ever‐demanding ivory market. As a result, their populations have fallen rapidly across their range (Hauenstein et al. 2019; Schlossberg et al. 2020). The situation has been further aggravated by loss of the elephant's habitat and its subsequent degradation (Sampson et al. 2021; Tomor 2015).

Across Africa, elephant populations have declined by over 60% (Chase et al. 2016), and in South Sudan by over 90% (Fay et al. 2007; Morjan et al. 2004). In Nimule National Park, South Sudan, the same has been observed as shown by the data collected using different methods, where it dropped by about 80% (Morjan et al. 2004; Sutherland 2006). From 1983 to 2004, a total of four studies were done. Kenyi (1983) and Abdalla (1988) each used both road and transect counts, resulting in 151 and 814 elephants, respectively. Morjan et al. (2000) applied dung counts, resulting in 156 elephants being counted. Morjan et al. (2004) employed both direct and dung counts and reported a population of 125 and 93 elephants, respectively. Though collected using different methods at different times and may not be reliable because, the population estimates obtained showed huge differences, the data indicate population decrease. Besides, for small populations, when using dung count method, only small numbers of dung piles will be observed along transects. As such, dung count will result in poor estimates that are to be of any significant use for assessing population trends (Hedges and Lawsons 2006); therefore, there is a need to investigate the genetic consequences of this decline. In addition, few investigators examined the degree of relatedness and kinship in the savannah elephant (Archie et al. 2006; Gobush et al. 2009). Moreover, studying the population genetics of the African elephant is necessary for its monitoring, conservation and management. Furthermore, it is important to examine whether the reported decline in elephant population in the park reflects the situation in the park or an artefact of the different population estimate methods used.

This study aimed to answer the following questions: first, how high is the level of genetic diversity of the savannah elephant population in the park? Second, did the elephant population in Nimule National Park experience a genetic bottleneck in the recent past? Third, how related are the elephants, or what is the level of kinship in the park? Fourth, are they genetically inbred?

## 2 Materials and Methods

### 2.1 Study Area

**FIGURE 1:**
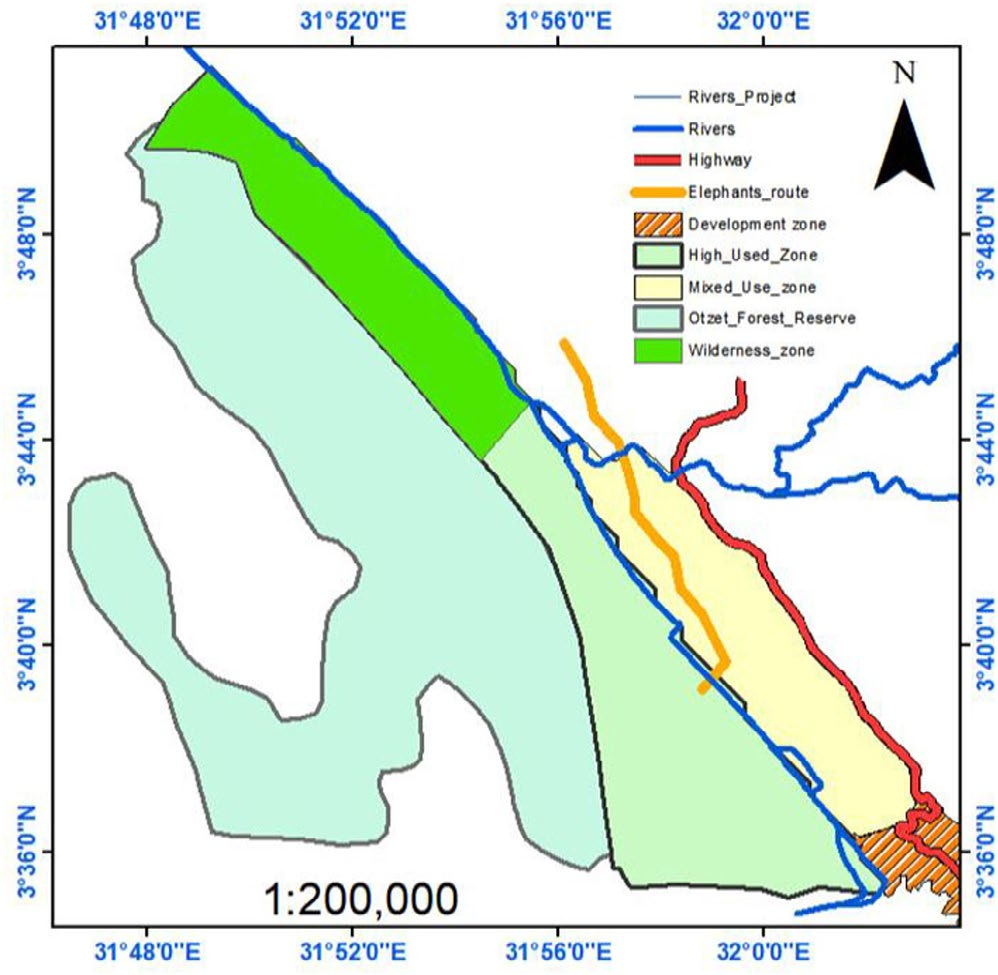
Map showing the location of Nimule National Park, South Sudan.

Nimule National Park is the smallest park in South Sudan. It was established to protect savannah elephant *Loxodonta africana africana* and the now locally extinct northern white rhino *Ceratotherium simum simum* . The park is situated at the end of the South Sudan–Uganda border between 03°50’N 31°30′ E (Figure 1). Its size is about 410 km^2^, including the buffer zone (Hillman 1985; BirdLife International 2017). The park is characterised by two seasons: a rainy season which starts in April and ends in November, and the dry season that runs from December to March. It has a mean annual rainfall of 1000–1200 mm and an average daily temperature of 27°C (Hillman 1985; van Noordwijk 1984). The vegetation of the park is predominantly a deciduous woodland savannah (Morjan et al. 2004; Van Noordwijk 1984). It is characterised by broad‐leafed and more foliage deciduous and evergreen trees with largely perennial grasses of up to about 3 m high (Morjan et al. 2004; Van Noordwijk 1984).

### 2.2 Field Sampling

Sampling of 5 g of fresh savannah elephant dung piles was done opportunistically along elephant trails for about 3 weeks in Nimule National Park, South Sudan. We covered about 25 trails, covering a total trail length of 90 km. Given an average trail width of 68 cm, this resulted in < 1% proportion of the study area being covered, which is small. However, elephant dung surveys recommend a total transect length of 75 km (Hedges and Lawsons 2006), and we covered more than the recommended length. Sampling was done in the rainy season, but during this time, climatic conditions in the park were dry and hot, a thing that is unusual for this area in the rainy season (Lado et al. 2024). At every encountered elephant dung pile, about 3 cm (5 g) of dung was collected from different sides and preserved in a vial containing 20% DMSO in Tris‐EDTA‐NaCl buffer at a pH > 10.0 according to the US permit number 45626 regulations. A total of 80 dung samples, at up to about 1 km apart from one another, were collected. Likewise, 20 samples of ivory collected from the ivory stockpile at the National Ministry of Wildlife Conservation and Tourism were collected, with each preserved and washed with 20% DMSO in Tris‐EDTA‐NaCl buffer at a pH > 10.0 for at least 2 h. Each of the dung and ivory samples was labelled according to sampling sequence, date and location, and then shipped to the United States in compliance with the US regulations for Veterinary Permit (permit number 45626) for Importation and Transportation of Restricted Substances and Organisms and Vectors.

### 2.3 Genomic DNA Extraction

Laboratory work was done at the “University of Washington, Conservation Biology Lab, Seatle, Washington, USA” following established protocols. Genomic DNA was extracted in duplicate from faecal samples using a QIA quick Stool DNA purification kit (Qiagen, Valencia, CA), followed by a Gene Clean III (Bio101) nucleic acid isolation kit for each template isolation. Negative controls were used for every 10 extractions accomplished. To identify and or minimise false alleles and allelic dropout (Fernando et al. 2003; Wasser et al. 2004; Wasser et al. 2007), dung extracts were diluted in multiples of 2, with PCR amplified with primers for the locus with the highest number of alleles (Fernando et al. 2003). Contamination of the PCRs was avoided by holding DNA extracts and PCR outfit in separate locations (Wasser et al. 2004). Multiple‐tubes approach and genotyping were used to provide more reliable genotyping of dilute DNA samples than a single tube approach (Fernando et al. 2003). Extracted genomic DNA from elephant dung each was amplified for 16 dinucleotide microsatellite loci following published protocols (Mailand and Wasser 2007; Wasser et al. 2004; Wasser et al. 2015). The resulting data were genotyped by two independent analysts calling allele sizes using Gene Marker v 2.4.0 (Soft Genetics) with a ~1000 relative fluorescent units (RFU) analysis threshold. To determine heterozygotes and homozygotes, all heterozygotes were counted twice, and homozygous individuals were recorded in a minimum of three PCR products from two independent DNA isolations derived from the same faecal sample (Wasser et al. 2004; Wasser et al. 2007), thereby enabling genotyping error rates to be estimated (Wasser et al. 2007). Such that when only one of the two alleles was confirmed, only that allele was incorporated and the other in the genotype was considered as “missing” where this is assumed to occur at random (Wasser et al. 2007).

### 2.4 Statistical Data Analysis

To determine the composition of the African elephants in the park, microsatellite samples derived from elephant dung were first analysed using assignment test based on their genotypes (Wasser et al. 2004). Second, a Markov chain Monte Carlo algorithm with spatial smoothing to compute separate continuous allele frequency maps across Africa from 1001 savannah elephant reference samples and 349 forest elephant reference samples collected across elephant range states was assigned to within 300 km accuracy (Wasser et al. 2015). After the assessment of the genetic composition of the elephant population in Nimule National Park, data on forest and hybrid elephants removed. Additionally, individual genotypes were profiled using allele frequencies across the 16 microsatellite loci studied (Comstock et al. 2002). Likelihood ratios were used to ascertain the probability of genotyped samples coming from the same or unique individuals after controlling for gene frequencies where the same individual matched across 13 of the 16 loci. Matched probabilities also controlled for the number of comparisons (Wasser et al. 2018; Wasser et al. 2022). These analyses culminated in savannah elephant data of 26 individuals upon which the following analyses were done. To determine the level of genetic variation within the savannah elephant population in the park, genetic diversity was quantified using standard summary statistics calculated for each locus and averaged over all loci. Analysis of genetic variation at a locus is important for identifying loci with the highest number of alleles or that are responsible for differences in a population (Akerman and Burger 2014). This was computed using Arlequin (Excoffier and Lischer 2010). To determine whether the elephant population in the park experienced a genetic bottleneck, two approaches were used. First, observed and expected heterozygosities employing the stepwise mutation model (SMM) and the two‐phase mutation model (TPM) (with the proportion of alleles attributed to SMM in TPM being 90% with a variance of 12) (Garza and Williamson 2001; Luikart et al. 1998; Piry et al. 1999) were calculated using BOTTLENECK software. Both SMM and TPM are considered the most appropriate mutation models for microsatellite data (Cornuet and Luikart 1996; Luikart and Cornuet 1998) with the TPM being more suitable in describing the mutational events in microsatellite loci (Kimura and Ohta 1978; Piry et al. 1999). The observed and expected heterozygosities were then compared using a one‐tailed Wilcoxon sign‐rank test that has high power with few individuals and polymorphic loci, with a significance level set at 5%. In addition, allele frequency distribution, which is considered to be more sensitive than heterozygosity and can detect recent reductions in population size (Cornuet and Luikart 1996; Luikart et al. 1998), was calculated using the same software, whereby in the case of no bottleneck, the allele frequency distribution is expected to be L‐shaped and skewed if there is a bottleneck both of these detect recent bottleneck. Second, M, the mean ratio between the number of alleles (k) and the range in allele size (r), where during a bottleneck incident, k decreases faster than r (Garza and Williamson 2001; Nei et al. 1975). It is considered to be more sensitive in uncovering a genetic bottleneck than for detecting a heterozygosity excess (Cornuet and Luikart 1996; Garza and Williamson 2001), and can detect past bottleneck events, was computed in Arlequin (Excoffier and Lischer 2010). Where M‐ratio of < 0.8 indicates the occurrence of a historical bottleneck (Garza and Williamson 2001). Relatedness was estimated using program RELATEDNESS 5.0 (Queller and Goodnight 1989) with the relatedness coefficient ML‐r weighted by individuals and standard errors obtained by jack‐knifing over populations. Kinship among the savannah elephants in Nimule National Park was determined using the genetic software ML‐RELATE (Kalinowski et al. 2006), which estimates the genetic relationships between all individual savannah elephants. The software estimates the maximum‐likelihood values of relatedness (ML‐r) and groups of pairwise relationships from genotypic data. These groups are U (unrelated), HS (half sibling), FS (full sibling) and PO (parent–offspring). To determine if the savannah elephant population in the park experienced inbreeding and was genetically structured, we used the AMOVA method implemented in Arlequin.

## 3 Results

### 3.1 Genetic Diversity

Standard measures of diversity were calculated for 26 savannah elephants dung samples and individuals, and the results are shown below (Table 1). Average allelic richness, observed (H_O_) and expected heterozygosity (H_e_) were 5.31 ± 2.62, 0.61 ± 0.226 and 0.56 ± 0.21 respectively. Observed (H_O_) and expected (H_e_) heterozygosities did not differ from each other, with Hardy Weinberg equilibrium across all loci and sampling sites being insignificant (*p* > 0.5). However, locus‐specific tests show that loci FH19, FH129, FH60 and FH103 disagreed with HWE (*p* < 0.05).

**TABLE 1:** Microsatellite loci used, number of gene copies, number of alleles, observed heterozygosity and expected heterozygosity.

Locus name	Number of gene copies	Number of alleles (A_o_)	Obs.Het. (H_o_)	Exp.Het. (H_e_)	G‐W stat
FH67	52	8	0.81	0.80	0.42
FH71	52	2	0.50	0.42	0.67
*FH19	52	7	0.77	0.66	0.47
*FH129	52	8	0.62	0.70	0.47
*FH60	52	3	0.23	0.21	0.60
FH127	51	9	0.80	0.81	0.07
FH126	52	8	0.88	0.75	0.47
FH153	51	8	0.88	0.79	0.38
FH94	51	5	0.52	0.56	0.22
FH48	52	4	0.77	0.63	0.02
FH40	50	2	0.36	0.30	0.67
FH39	52	9	0.77	0.82	0.04
*FH103	52	4	0.35	0.31	0.44
FH102	52	3	0.42	0.36	0.60
SO3	52	2	0.31	0.27	0.67
SO4	52	4	0.69	0.63	0.57
Mean	51.69	5.38	0.61	0.56	0.42
SD (±)	0.58	2.6	0.22	0.21	0.22

*Note*: Line 3 of the results narrative, expected heterozygosity (He) should be 0.61 ± 0.22 and not 0.61 ± 0.226.

*Indicates loci that do not conform to Hardy‐Weinberg expectations.

### 3.2 Bottleneck

**FIGURE 2:**
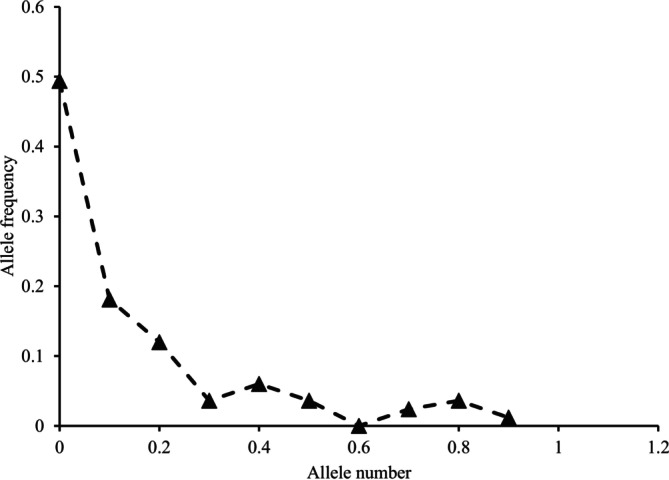
L‐shaped allele frequencies mode shift in savannah elephant population in Nimule National Park.

Under SMM, there was no significant difference between the observed and the expected heterozygosities (*p* = 0.9416). Likewise, under the TPM model, the result shows no significant heterozygosity excess (*p* = 0.98550). The mode shift of allele frequencies is L‐shaped, indicating no evidence of a recent genetic bottleneck (Figure 2).

However, the M‐ratio bottleneck test showed signals of demographic bottleneck in the past. The M‐ratio bottleneck test detects a drop in allele size range for all loci and individuals showing M‐ratios equal to 0.44 ± 0.22.

### 3.3 Relatedness, Kinship and Inbreeding

Mean pairwise relatedness was generally low at 9.4% (ML‐*r* = 0.09 ± 0.22). The proportion of unrelated individuals was the highest (U = 85.8%), and full siblings were the least (FS = 1.5%). The inbreeding coefficients estimated for this population were low and not significant (F_IS_ = −0.08, *p* > 0.05), suggesting no inbreeding in the savannah elephant population in Nimule National Park (Table 2).

**TABLE 2:** Relatedness, kinship and inbreeding in savannah elephant population in Nimule National Park.

Genetic measure	Per cent value
**Pairwise maximum‐likelihood relatedness (ML‐r)**	
Mean ML‐r (± SD)	0.09 (± 0.22)
**Kinship**	
Unrelated individuals (U)	86% (279/325)
Half siblings (HS)	10% (31/325)
Full siblings (FS)	2% (5/325)
Parent–offspring (P/O)	2% (10/325)
**Inbreeding**	
F_IS_	–0.08 (*p* > 0.05)

## 4 Discussion

### 4.1 Level of Genetic Diversity

Observed H_o_ and H_e_ expected heterozygosity were 0.61 ± 0.22 and 0.56 ± 0.21, respectively, with no difference between them and observed allelic richness of A_o_ = 5.31 ± 2.62. The observed level of genetic variation in the study area may be attributed to the presence of individuals possessing novel alleles in the savannah elephant population studied here, possibly due to admixture. Elephants are reported to move between Nimule National and Otze Forest in Uganda (F. Michelmore, pers. Comm., 1998, mentioned in Blanc et al. 2003), and between Yei, Kajo Keji, Nimule and Moyo (11 miles from Nimule) (Reid 1952). Undeniably, when genetic subpopulations of the same species mate, it can lead to a surge in genetic variation (Alleaume‐Benharira et al. 2006; Hartl and Clark 1997). Compared to all the savannah elephant populations genetically studied to date across Africa, average H_e_ across loci was higher in Kenya (H_e_ = 0.75, Okello et al. 2008), in Kavango‐Zambezi Transfrontier Conservation Area (H_e_ = 0.71, De Fleming 2013), Greater Kruger Biosphere (H_e_ = 0.64, Santos et al. 2019) and Tanzania (H_e_ = 0.73, Lohay et al. 2020) than that obtained in this study (H_e_ = 0.56). However, the average expected heterozygosity reported for this study is higher than that reported for the savannah elephant population of the Gash‐Barka in Eritrea (H_e_ = 0.29, Brandt et al. 2014).

### 4.2 Bottleneck

No evidence for a recent genetic bottleneck (*p* = 0.94167, and Normal L‐shaped distribution), but a historical bottleneck was detected (M‐ratio = 0.44 ± 0.22). The lack of evidence for a recent genetic bottleneck, as indicated by the TPM mutation model and the normal L‐shaped allele frequency distribution, is most likely due to population admixture (Frankham et al. 2010; McEachern et al. 2011). Also, it may be due to the time and length of the population decline, as well as the level of genetic diversity present in the savannah elephant population in the park before and after the bottleneck (Busch et al. 2007; Kramer and Sarnelle 2008; McEachern et al. 2011; Williamson‐Natesan 2005). This result is not unique to this study. Bottleneck tests did not detect signatures of a recent bottleneck in savannah elephant populations in Queen Elizabeth, Murchison Falls and Kidepo Valley National Parks in Uganda (Muwanika et al. 2003) and in the Greater Kruger Biosphere in South Africa (Santos et al. 2019). Our study agreed with both authors on one factor responsible for the failure to detect a recent bottleneck in the studied population as a result of immigration from the nearby populations. However, our study differed with these authors who besides immigration attributed the lack of bottleneck to other additional factors. Muwanika et al. (2003) stated the possibility of the observed excess heterozygosity in post‐bottleneck populations being a transient feature, and therefore expected to last only a few generations, as another factor. Santos et al. (2019) listed demographic recovery, large pre‐bottleneck population size and the long elephant generation time. Remarkably, the indication by the Garza–Williamson index that a bottleneck had occurred in the savannah elephant population in Nimule National Park can be associated with the increasing demand for ivory and the heavy, unregulated poaching of elephants in the area extending between central South Sudan and northern Uganda in the 19th century (Happold 1966; Leopold 2009; Owen 1949). Our results, indicating a historical bottleneck, disagreed with Santos et al. (2019), who did not find a historical bottleneck (Garza‐Williamson M = 0.9) in the Greater Kruger Biosphere Reserve in South Africa and attributed it to the aforementioned factors.

### 4.3 Relatedness and Kinship

Mean pairwise relatedness was generally low (ML‐*r* = 0.09 ± 0.22) with a high proportion of unrelated individuals (U = 85.8%). Generally, levels of relatedness at the 16 polymorphic microsatellite loci in the studied savannah elephant population were low and comparatively lower than those reported for the elephant population in Amboseli (ML‐*r* = 0.15 ± 0.2, Archie et al. 2006) and Mikumi (ML‐*r* = 0.13 ± 0.1, Gobush et al. 2009). The negative F_IS_ may be due to the presence of a high proportion of unrelated individuals. Immigration of unrelated individuals can thwart higher levels of inbreeding (Allendorf 1986; Nei et al. 1975). Additionally, the observed low mean genetic relatedness, along with the high percentage of unrelated individuals, demonstrates that the studied savannah elephant population in Nimule National Park contains many distantly related individuals. In addition, this may suggest that the savannah elephant population in the park was a mix of individuals, for example, from poaching‐disrupted social groups (Gobush et al. 2009; Gobush and Wasser 2009). This is because elephants usually live in social groups characterised by high mean relatedness (Archie et al. 2006; Charif et al. 2005; Nyakaana et al. 2001). Furthermore, although the sex of the 26 individuals was not determined, nevertheless authors during fieldwork have encountered groups of elephants with young foraging together. Additionally, one dung sample was found to belong to a hybrid elephant. These, though not supported by sex data of the individuals, point to the possible effect of poaching or anthropogenic pressure. Moreover, conflicts over resources and mates, as well as increased levels of human–elephant conflict, have been observed.

### 4.4 Inbreeding

The elephant population was not inbred (F_IS_ = −0.08, *p* > 0.05). The positive effect of population admixture on genetic variability is the introduction of unique alleles into the genetic pool of the recipient population, thereby enhancing heterozygosity and counteracting inbreeding (Frankham et al. 2010; Keller and Waller 2002). Our study agrees with other investigators who did not find signs of inbreeding in populations of the savannah elephant from Eastern and southern Africa (Nyakaana et al. 2002) and from Eritrea (Brandt et al. 2014). However, our study disagrees with Santos et al. (2019), who found signatures of inbreeding in the savannah elephant population from Greater Kruger Biosphere, South Africa. Although the latter author suggested that the observed inbreeding in the Greater Kruger Biosphere savannah elephant population may be due to null alleles, allelic dropout and fine‐scale genetic structure rather than as a result of mating between relatives. There are several reasons underlying the lack of inbreeding in these populations. These reasons may be different for each population. The lack of inbreeding in the populations is plausible given that savannah elephant populations have a long generation time, and since the number of mutations inherited by an offspring is influenced by the number of parental germline cell divisions; and germline divisions increase with generation time, then the longer the generation time, the more diversity (see Bronham 2009; Latta IV et al. 2003). Besides, immigration into and, inconsequently, gene flow from the immigrants into the resident savannah elephant population in Nimule National Park might have prevented the reduction in effective population size in Nimule National Park from being detected (Hall‐Martin 1992; Frankham et al. 2010). In addition, the post‐bottleneck population recovery of the savannah elephant might have been rapid for the available tests to detect the occurrence of a bottleneck (Busch et al. 2007; Okello et al. 2008). However, for our case, the most likely reason is immigration into and gene flow from the immigrants into the resident savannah elephant population in Nimule National Park might have prevented the reduction in effective population size in Nimule National Park from being detected (Hall‐Martin 1992; Frankham et al. 2010).

### 4.5 Implications

The conservation implications of this study were; first, the observed genetic diversity shows that the savannah elephant population in the park may not be at imminent risk as it appears to be receiving immigrants from other populations in the surrounding areas. Second, the savannah elephant population shows no sign of inbreeding, along with the low genetic relatedness, which may suggest that at least in the short term, inbreeding and inbreeding depression do not constitute a conservation concern, and gene flow might be acting in the population. Although these may seem assured, there is a need for further research for a better understanding of genetic diversity, demographic characteristics and relatedness of the nearby populations of savannah elephants to establish if the Nimule population of savannah elephants is part of one big population for Nimule National Park and the nearby areas known to harbour savannah elephant populations. Second, radio‐collaring of the elephants is needed to determine the distance and places they go to, with the aim of conserving those areas which contain critical habitat for the elephant and safeguarding the routes used by them so as to minimise human–elephant conflict, which is a serious problem in the areas surrounding the park.

## Author Contributions

**Thomas Francis Lado:** conceptualization (equal), data curation (lead), formal analysis (lead), writing – review and editing (lead). **Wani Felix Jibi:** conceptualization (supporting), data curation (supporting). **Pasquale Tiberio Moilinga:** resources (supporting).

## Conflicts of Interest

The authors declare no conflicts of interest.

## Data Availability

The data that supports the findings of this study are available in the supplementary material of this article.
